# Amyloid Myopathy: A Cunning Masquerader

**DOI:** 10.7759/cureus.39576

**Published:** 2023-05-27

**Authors:** Guru Prasad Parthiban, Jon Wilson, Joseph P Nesheiwat

**Affiliations:** 1 Internal Medicine, Baton Rouge General, Baton Rouge, USA; 2 Pathology, Arkana Laboratories, Little Rock, USA; 3 Rheumatology, Baton Rouge Clinic, Baton Rouge, USA

**Keywords:** al amyloid, monoclonal gammopathy, immunoglobulin light-chain amyloidosis, pelvic girdle myopathy, systemic amyloid

## Abstract

Amyloid myopathy (AM) is a rare manifestation of systemic amyloidosis (AL) or isolated amyloid myopathy, based on which the clinical features can vary. AM can have overlapping features with idiopathic inflammatory myopathies, and a muscle biopsy with Congo red staining is essential to differentiate between both. Other investigations, including a comprehensive myositis panel, magnetic resonance imaging (MRI) of the involved muscle group, and echocardiography, can also be beneficial. Treatment is based on the type of amyloid protein deposited and other organ involvement. This article reports a 74-year-old female with multiple features suggestive of antisynthetase syndrome, which, upon further workup, was proven to be a challenging case of amyloid myopathy secondary to immunoglobulin light chain AL.

## Introduction

Amyloidosis (AL) is an infiltrative disorder characterized by the deposition of insoluble fibrous proteins called amyloids in tissues and multiple organs, causing damage. To date, up to 36 amyloidogenic proteins have been identified, based on which amyloidosis is classified into subtypes. Most common subtypes include immunoglobulin light-chain or primary AL, AA amyloidosis secondary to chronic inflammation, familial or mutated transthyretin (ATTR) amyloidosis, wild-type or senile amyloidosis, and amyloidosis associated with genetically mutated fibril protein precursors such as cystatin and gelsolin [1]. The presentation of clinical features usually depends on the organ involved. Primary amyloidosis presenting as amyloid myopathy (AM) is extremely rare, with an incidence of 1.5% [2]. The diagnosis is easily missed or misdiagnosed as one of the inflammatory myopathies, as many clinical symptoms and even muscle biopsy findings can overlap.

## Case presentation

A 74-year-old female patient with a history of Raynaud’s syndrome and bilateral carpal tunnel syndrome presented with progressively worsening pain involving multiple joints and muscles of one-month duration. The pain was predominant in her shoulders and thighs and severe enough that she could not perform daily activities without assistance. Shortness of breath was exertional and relieved with rest. The cough was productive, with white sputum. Other reviews of systems were negative for fever, rashes, dysphagia, chest pain, recent sick contact, nausea, vomiting, or diarrhea. The review of the medication was negative for offending agents. No history of recreational drug use or alcohol. She had significant proximal muscle weakness and tenderness involving her arms and thighs on examination. Initial lab work is noted in Table 1.

**Table 1:** Initial laboratory results

Labs	Results (units/liter)
Aspartate aminotransferase (AST)	1020
Alanine transaminase (ALT)	497
Alkaline phosphatase (ALP)	40
Creatine kinase	>14,000
Aldolase	180.6
LDH	2444

**Figure 1:**
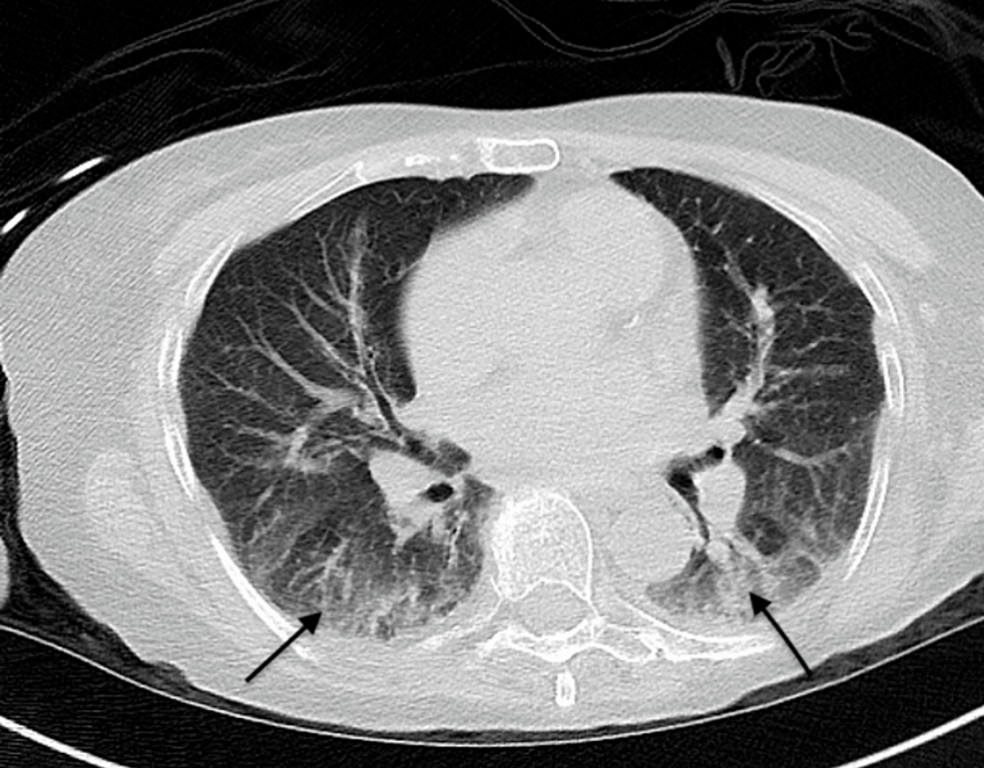
CT chest showing bilateral basal atelectasis

Chest X-ray and computed tomography (CT) of the chest revealed basal atelectasis but were not definitive in excluding interstitial lung disease (ILD) (Figure 1).

Suspicion for immune-mediated myopathy was high, and an anti-nuclear antibody (ANA) panel was ordered. ANA was negative, but the cytoplasmic signal was present on fluorescent ANA. A comprehensive myositis panel was positive for anti-Ro/SSA-52 kD antibodies. At this point, a clinical diagnosis of antisynthetase syndrome was favored; however, antisynthetase antibodies were negative on the myositis panel (Table 2).

**Table 2:** Comprehensive myositis panel

Antibody	Results
Antisynthetase antibodies (Jo-1, PL-7, PL-12, EJ, OJ)	Negative
Anti-SRP (necrotizing myositis)	Negative
Anti-Mi2	Negative
Anti-MDA5	Negative
Anti-SSA/Ro52	Positive

**Figure 2:**
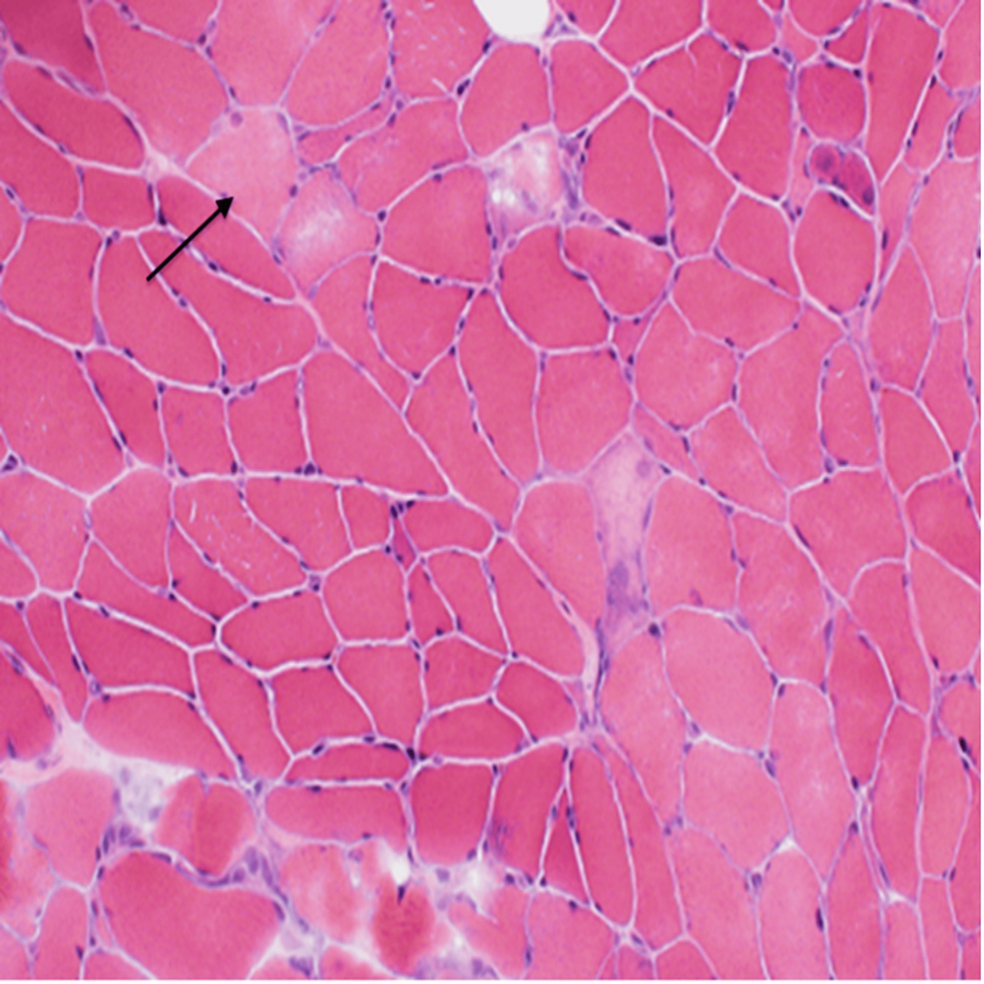
Muscle biopsy showing regenerating muscle fibers without evidence of myonecrosis

For confirmation of the diagnosis, a muscle biopsy was obtained (Figure 2), which showed regenerating muscle fibers without evidence of myonecrosis.

**Figure 3:**
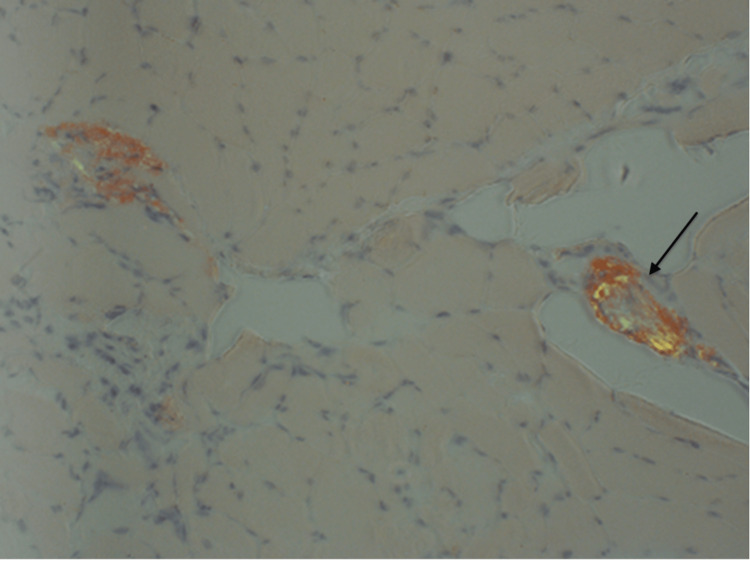
Polarized light imaging demonstrating apple-green birefringence of vascular amyloid deposits

Congo-Red staining of muscle biopsy tissue revealed vascular amyloid deposition, which showed apple-green birefringence under polarized light (Figure 3).

**Figure 4:**
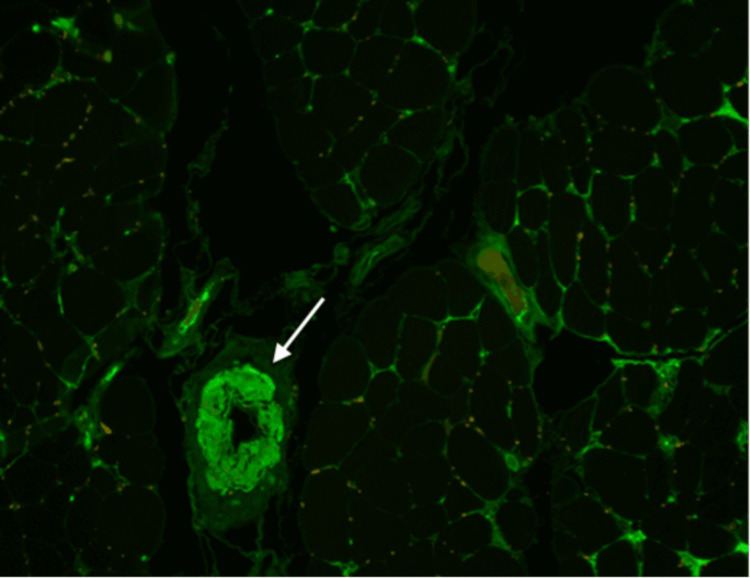
Immunofluorescence microscopy with positive lambda light chain

Immunofluorescence microscopy was positive for the lambda light chain (Figure 4).

Immunofixation studies confirmed monoclonal IgA with the lambda light chain. She has been initiated on prednisone 60 mg daily, with improvement in her myalgia. She was discharged to a rehabilitation center for continuing physical therapy. She was also referred to the Hematology/Oncology Outpatient Service for further evaluation of her IgA gammopathy.

## Discussion

Amyloid myopathy is defined by the presence of amyloid deposits in the intramuscular blood vessels or connective tissue of the skeletal muscle. It is important to note that these amyloid deposits are extracellular, whereas amyloid deposited in other myopathies, such as inclusion body myopathies, is intracellular and confined to the sarcoplasm [3]. AM could be secondary to systemic amyloidosis or isolated amyloid myopathy, based on which the presenting symptoms can vary. It is also essential to distinguish amyloid myopathy from idiopathic inflammatory myopathies (IIM), as they have overlapping clinical features and require muscle biopsy with Congo red staining to differentiate between both.

AL amyloidosis is the most common form of systemic amyloidosis and accounts for about 61% of total amyloid myopathy cases, per the study by Liewluck and Milone [3]. The typical presentation of AL amyloidosis includes but is not limited to restrictive cardiomyopathy, organomegaly, peripheral neuropathy, and nephrotic syndrome. The neuropathy usually involves the smaller sensory and autonomic fibers initially, and the patients presents with pain and paresthesia in the feet. As the disease progresses, larger sensory and motor nerves can also be involved. Patients can also have autonomic dysfunction such as orthostatic hypotension, sweating disorders, erectile dysfunction, dry eyes, etc. Jaw claudication can also occur in 9-25% of patients with AL amyloidosis. Macroglossia and periorbital purpura are other features specific to AL amyloidosis [2]. Occasionally, patients can have amyloid depositions in their joints, including shoulder capsules which can cause joint stiffness and affect mobility. This leads to loss of muscle mass in the shoulder girdle and makes the shoulder fat pad more prominent, a characteristic sign of AL amyloidosis. About 50% of AL amyloidosis patients have bilateral carpal tunnel syndrome that can precede the diagnosis by two years [4].

Isolated amyloid myopathy without systemic amyloidosis is very rare and is reported to be secondary to recessive mutations in a gene called anoctamin-5 (ANO5). Some characteristic features of patients with this mutation include asymmetric weakness and atrophy of the thigh and calf muscles, myalgia, rhabdomyolysis, and asymptomatic CK elevations several years before symptoms onset [5]. There are additional reports of isolated amyloid myopathy secondary to dysferlin (DYSF) gene mutations causing limb girdle muscular atrophy [6]. Usually, patients with isolated amyloid myopathy tend to be younger, with a median onset age of 41.5 years, whereas it is 65.5 years in the case of AL amyloidosis with skeletal muscle involvement. Median CK levels tend to be higher in the isolated amyloid group when compared to AL amyloidosis (8.4 vs. 0.77, respectively) [3].

MRI can be a useful diagnostic tool in AM. Per Metzler et al., who reported 2 AM patients, MRI showed prominent reticulation of subcutaneous fat and negligible signal intensity alteration of the muscle. This finding was found to be in contrast with other varieties of inflammatory myopathies in which MRI is prominent with muscle edema or fatty changes of the muscle [7]. It has been estimated that at least 67% of patients with AL amyloidosis have echocardiographic abnormalities such as abnormal myocardial relaxation, thickened ventricular walls, pericardial effusion, and a restrictive filling pattern [4]. Electromyography (EMG) is not routinely performed, but it can show low amplitude action potentials with rapid recruitment or sometimes fibrillation potentials in certain patients. A muscle biopsy is necessary to confirm the diagnosis and for amyloid subtyping. In inflammatory myopathies such as polymyositis, endomysial inflammation by lymphocytes and macrophages infiltrating the non-necrotic muscle fibers is characteristic. A biopsy can be obtained from the minor salivary glands (86% sensitivity) or the deep rectal submucosa (75-85% sensitivity) [4]. Necrosis, regeneration, and atrophy are nonspecific findings. Our patient had similar nonspecific findings of non-necrotic regenerating muscle fibers. But when we used Congo-Red staining and polarized light exam, amyloid deposition in vessel walls with apple green birefringence was apparent. This is a diagnostic finding in amyloidosis. Once amyloid is confirmed in the biopsy, it is ideal to perform subtyping with mass spectrometry, which has high sensitivity and specificity.

In general, treatment is tailored based on the severity of organ involvement to reduce amyloid light chain levels. Autologous stem cell transplant (ASCT) is the gold standard treatment for systemic amyloidosis. Patients who are not candidates for ASCT are usually treated with disease-modifying therapies such as bortezomib, daratumumab, pomalidomide, and venetoclax. Combination therapy with bortezomib in addition to melphalan and/or cyclophosphamide carries a better prognosis, and upfront administration of bortezomib led to a prolonged hematological response [1]. For ATTR amyloidosis-induced cardiomyopathy, tafamidis, a transthyretin stabilizer, is approved for use. Some countries, such as Europe and South America, have also approved Tafamidis for ATTR amyloidosis-induced polyneuropathy [2]. The prognosis depends on the type of amyloid protein deposited and the organ involved. AL amyloidosis carries the worst prognosis, and patients with heart failure have the lowest median survival of about six months [4].

## Conclusions

In summary, our patient had high clinical suspicion for inflammatory myopathy secondary to the antisynthetase syndrome, given the patient’s elevated CK, abnormal lung CT concerning interstitial lung disease, cytoplasmic signal on fluorescent ANA, and Ro-52 kD antibody (myositis-associated antibody). Given this constellation of findings, it is easy to confine the diagnosis to the antisynthetase syndrome. Since the diagnosis was not definitive, a muscle biopsy was obtained, which changed the course of her management. It is also essential to incorporate the routine use of the Congo-Red staining technique in all diagnostic muscle biopsy specimens, especially in the elderly population, which increases the frequency of the diagnosis of amyloid myopathy.
